# Outcomes of etravirine-based antiretroviral treatment in treatment-experienced children and adolescents living with HIV in Europe and Thailand

**DOI:** 10.1177/13596535221092182

**Published:** 2022-06-01

**Authors:** European Pregnancy and Paediatric Infections Cohort Collaboration (EPPICC), Alex Lyons, Lindsay Thompson, Elizabeth Chappell, Luminita Ene, Luisa Galli, Tessa Goetghebuer, Gonzague Jourdain, Antoni No-Guera-Julian, Christian R. Kahlert, Christoph Königs, Pope Kosalaraksa, Pagakrong Lumbiganon, Magdalena Marc-Zynska, Laura Marques, Marissa Navarro, Lars Naver, Liubov Okhonskaia, Filipa Prata, Thanyawee Puthanakit, Jose Tomas Ramos, Anna Samarina, Claire Thorne, Evgeny Voronin, Anna Turkova, Carlo Giaquinto, Ali Judd, Intira J Collins, Alex Lyons, Lindsay Thompson, Elizabeth Chappell, Luminita Ene, Luisa Galli, Tessa Goetghebuer, Gonzague Jourdain, Antoni Noguera-Julian, Christian R Kahlert, Christoph Königs, Pope Kosalaraksa, Pagakrong Lumbiganon, Magdalena Marczyńska, Laura Marques, Marissa Navarro, Lars Naver, Liubov Okhonskaia, Filipa Prata, Thanyawee Puthanakit, Jose T Ramos, Anna Samarina, Claire Thorne, Evgeny Voronin, Anna Turkova, Carlo Giaquinto, Ali Judd, Intira J Collins

**Affiliations:** MRC Clinical Trials Unit at UCL, University College London, London, UK; MRC Clinical Trials Unit at UCL, University College London, London, UK; MRC Clinical Trials Unit at UCL, University College London, London, UK; Clinical Department of Infectious Diseases (HIV Department), Dr. Victor Babes¸ Hospital for Infectious and Tropical Diseases, Bucharest, Romania; Infectious Disease Unit, Meyer Children's Hospital, Department of Health Sciences, University of Florence, Florence, Italy; Hopital St Pierre, Brussels, Belgium; Institut de recherche pour le développement (IRD)-PHPT, Marseille, France; Faculty of Associated Medical Sciences, Chiang Mai University, Chiang Mai, Thailand; Infectious Diseases and Systemic Inflammatory Response in Pediatrics, Infectious Diseases Unit, Department of Pediatrics, Sant Joan de Déu Hospital Research Foundation, Barcelona, Spain; Children’s Hospital of Eastern Switzerland and Cantonal Hospital, Infectious Diseases and Hospital Epidemiology, St Gallen, Switzerland; Department for Children and Adolescents, Division for Stem Cell Transplantation and Immunology, University Hospital Frankfurt/Main, Germany; Department of Pediatrics, Khon Kaen University, Khon Kaen, Thailand; Department of Pediatrics, Khon Kaen University, Khon Kaen, Thailand; Medical University of Warsaw, Hospital of Infectious Diseases, Warsaw, Poland; Centro Hospitalar e Universitário do Porto, Porto, Portugal; Hospital General Universitario “Gregorio Marañón”, Madrid, Spain; Universidad Complutense, Madrid, Spain; Instituto de Investigación Sanitaria Gregorio Marañón (IISGM), Spain; Red de Investigación Translacional en Infectología Pediátrica, RITIP, Madrid, Spain; Karolinska University Hospital and Karolinska Institutet, Stockholm, Sweden; Federal Budgetary Institution “Republican Clinical Infectious Hospital” of the Ministry of Health of the Russian Federation, Saint-Petersburg, Russian Federation; Hospital de Santa Maria/CHULN, Lisbon, Portugal; Department of Pediatrics, Faculty of Medicine, Chulalongkorn University and HIVNAT, Thai Red Cross AIDS Research Center, Bangkok, Thailand; Departamento de Salud Pública y Materno-infantil. Universidad Complutense. Hospital Clínico San Carlos, Instituto de Investigación Sanitaria del Hospital Clínico San Carlos (IdISSC), Madrid. Spain; The City HIV centre, St. Petersburg City AIDS Center, St Petersburg, Russian Federation; University College London Great Ormond Street Institute of Child Health, London, United Kingdom; Federal Budgetary Institution “Republican Clinical Infectious Hospital” of the Ministry of Health of the Russian Federation, Saint-Petersburg, Russian Federation; MRC Clinical Trials Unit at UCL, University College London, London, UK; Great Ormond Street Hospital, London, UK; Paediatric European Network for the Treatment of AIDS (Penta), Padova, Italy; MRC Clinical Trials Unit at UCL, University College London, London, UK; MRC Clinical Trials Unit at UCL, University College London, London, UK; 1 MRC Clinical Trials Unit at UCL, University College London, London, UK; 2 Clinical Department of Infectious Diseases (HIV Department), Dr. Victor Babes¸ Hospital for Infectious and Tropical Diseases, Bucharest, Romania; 3 Infectious Disease Unit, Meyer Children’s Hospital, Department of Health Sciences, University of Florence, Florence, Italy; 4 Hopital St Pierre, Brussels, Belgium; 5 Institut de recherche pour le développement (IRD)-PHPT, Marseille, France; 6 Faculty of Associated Medical Sciences, Chiang Mai University, Chiang Mai, Thailand; 7 Infectious Diseases and Systemic Inflammatory Response in Pediatrics, Infectious Diseases Unit, Department of Pediatrics, Sant Joan de Déu Hospital Research Foundation, Barcelona, Spain; 8 Infectious Diseases and Hospital Epidemiology, Children’s Hospital of Eastern Switzerland and Cantonal Hospital, St Gallen, Switzerland; 9 Department for Children and Adolescents, Division for Stem Cell Transplantation and Immunology, University Hospital Frankfurt/Main, Frankfurt, Germany; 10 Department of Pediatrics, Khon Kaen University, Khon Kaen, Thailand; 11 Hospital of Infectious Diseases, Medical University of Warsaw, Warsaw, Poland; 12 Centro Hospitalar e Universitário do Porto, Porto, Portugal; 13 Hospital General Universitario “Gregorio Marañón”, Madrid, Spain; 14 Universidad Complutense, Madrid, Spain; 15 Instituto de Investigación Sanitaria Gregorio Marañón (IISGM), Madrid, Spain; 16 Red de Investigación Translacional en Infectología Pediátrica, RITIP, Madrid, Spain; 17 Karolinska University Hospital and Karolinska Institutet, Stockholm, Sweden; 18 Federal Budgetary Institution “Republican Clinical Infectious Hospital” of the Ministry of Health of the Russian Federation, Saint-Petersburg, Russian Federation; 19 Hospital de Santa Maria/CHULN, Lisbon, Portugal; 20 Department of Pediatrics, Faculty of Medicine, Chulalongkorn University and HIVNAT, Thai Red Cross AIDS Research Center, Bangkok, Thailand; 21 Departamento de Salud Pública y Materno-infantil, Universidad Complutense, Hospital Clínico San Carlos, Instituto de Investigación Sanitaria del Hospital Clínico San Carlos (IdISSC), Madrid, Spain; 22 The City HIV Centre, St Petersburg City AIDS Center, St Petersburg, Russian Federation; 23 University College London Great Ormond Street Institute of Child Health, London, UK; 24 Great Ormond Street Hospital, London, UK; 25 Paediatric European Network for the Treatment of AIDS (Penta), Padova, Italy

## Abstract

**Background:**

Etravirine (ETR) is approved as a component of second or third-line antiretroviral treatment (ART) for children living with HIV. We assessed the outcomes of ETR-based ART in children in routine care in Europe and Thailand.

**Methods:**

Data on children aged <18 years at ETR start were pooled from 17 observational cohorts. Characteristics at ETR start, immunological and virological outcomes at 12 months, discontinuations, adverse events (AEs) and serious adverse events (SAEs) were described. Follow-up was censored at ETR discontinuation, death or last visit.

**Results:**

177 children ever received ETR. At ETR start, median [IQR] age was 15 [12,16] years, CD4 count 480 [287, 713] cells/mm^3^, 70% had exposure to ≥3 ART classes and 20% had viral load (VL) <50 copies/mL. 95% received ETR in combination with ≥1 potent drug class, mostly protease inhibitor-based regimens. Median time on ETR was 24 [7, 48] months. Amongst those on ETR at 12 months (*n*=141), 69% had VL<50 copies/mL. Median CD4 increase since ETR start (*n*=83) was 147 [16, 267] cells/mm^3^. Overall, 81 (46%) discontinued ETR by last follow-up. Median time to discontinuation was 23 [8, 47] months. Common reasons for discontinuation were treatment simplification (19%), treatment failure (16%) and toxicity (12%). Eight children (5%) had AEs causally associated with ETR, all dermatological/hypersensitivity reactions. Two were SAEs, both Stevens–Johnson Syndrome in children on regimens containing ETR and darunavir and were causally related to either drugs; both resolved following ART discontinuation.

**Conclusion:**

Children receiving ETR were predominantly highly treatment-experienced, over two-thirds were virally suppressed at 12 months.

## Introduction

Second- and third-line antiretroviral treatments (ART) are needed for children and adolescents living with HIV who experience treatment failure and have limited ART options, due to multidrug resistance or drug intolerance [1–3]. Etravirine is a second-generation non-nucleoside reverse-transcriptase inhibitor (NNRTI) approved for use as part of second or third-line ART in children and adolescents aged ≥6 years [4]. Approval was extended to children ≥2 years weighing ≥10 kg in the USA in 2018 [5] and in Europe in 2020 [4]. ETR is included as a potential component of second and subsequent line regimens in previous and current international paediatric HIV treatment guidelines [6–8].

Clinical outcome data for ETR in treatment-experienced children and adolescents are limited to single-arm trials and observational studies with <50 patients. These reported 33%–78% viral suppression <50 copies/mL at 48 weeks [9–14]. Common adverse reactions included gastrointestinal disorders and rashes. This study pooled data from the European Pregnancy and Paediatric Infections Cohort Collaboration (EPPICC) to assess the characteristics, effectiveness and safety of ETR in routine care across Europe and Thailand.

## Methods

Individual patient data were pooled from 17 cohorts from 11 European countries and Thailand, for children/adolescents starting ETR aged <18 years, with followed-up data until 31/01/2018. Individual cohorts gained local ethics approval. Data were collected using the HIV Cohort Data Exchange Protocol (www.hicdep.org) and included demographics, clinical and ART history, CD4, viral load (VL), biochemistry results and adverse events (AEs).

Children were categorised into five mutually exclusive groups based on age, treatment history and initial ETR dose for weight: (1) licenced; (2) unlicensed; (3) off-label, ART inexperienced (no prior protease inhibitor (PI) or NNRTI exposure); (4) off-label, age < 6 years; and (5) missing weight/dose. Treatment-experienced children ≥6 years, receiving an ETR dose within ±20% of European dose recommendations for weight at ETR start (twice daily ETR dosing: 100 mg (≥16 to <20 kg), 125 mg (≥20 to <25 kg), 150 mg (≥25 to <30 kg) and 200 mg (≥30 kg)) were as-signed to the ‘licenced’ group [4].

Follow-up time in ETR-related clinical trials was excluded. Characteristics at ETR start and frequency and reasons for ETR discontinuation were described. Amongst those on ETR at 12 months, immunological response (change in CD4 cell count) and virological suppression (VL<50 & <400 copies/mL) were summarised. Rates of Division of AIDS (DAIDS) [15] grade ≥3 laboratory AEs were estimated for the licenced dose group (all other groups had sample sizes <20), for absolute neutrophil count (ANC), total cholesterol, triglycerides, alanine transaminase (ALT), total bilirubin, fasting and non-fasting plasma glucose, pancreatic amylase, pancreatic lipase, aspartate amino-transferase (AST), alkaline phosphatase (ALP) and low-density lipoprotein cholesterol (LDL). Rates were reported overall and at <12, 12–24 and >24 months after ETR start. Follow-up time for laboratory AEs was censored at first AE within each time-period or 30 days after ETR discontinuation. Clinical AEs and serious AEs (SAEs) were described and rates of specific events reported. Follow-up time was censored at the earliest of: discontinuation of ETR, death or last visit. STATA version 16 (College Station, TX: StataCorp LLC) was used for all analyses.

## Results

Of 182 children who ever received ETR, five were excluded as all time on ETR was within a clinical trial. Of 177 remaining, half were male and 98% acquired HIV perinatally. At ETR start, median [IQR] age was 15 [12,16] years and 99% were treatment-experienced (Table 1). Median time since ART initiation was 11 [8,13] years and 70% had prior triple class exposure (NRTI, NNRTI and PI). At ETR start, 45% had a prior CDC C diagnosis, median CD4 was 480 [287,713] cells/mm^3^ and 20% had VL<50 copies/mL. Nearly all patients (95%, 169/177) started ETR in combination with another potent drug class: 100 (56%) with a PI-based regimen, 7 (4%) integrase inhibitor (INI)-based regimen and 62 (35%) with multiple drug classes (PI, INI, fusion inhibitor and/or CCR5 inhibitor). Among those receiving a PI, the most common was darunavir (84% (136/162)). Overall, 61% started ETR on a licenced dose, 6% unlicensed dose, 4% off-label as ART inexperienced, 3% off-label as aged <6 years (median age 4.5 [2.2, 5.0] years) and 26% had missing weight/dose.

Median follow-up after ETR initiation was 24 [7, 48] months. Amongst patients on ETR at 12 months (*n*=141), 69% (85/124 with data) had VL<50 copies/mL and 80% (99/124) <400 copies/mL, median CD4 (*n*=103) was 658 [454, 853] cells/mm^3^ and median CD4 change from ETR start (*n*=83) was 147 [16, 267] cells/mm^3^ (*p*<0.001).

**Figure 1:**
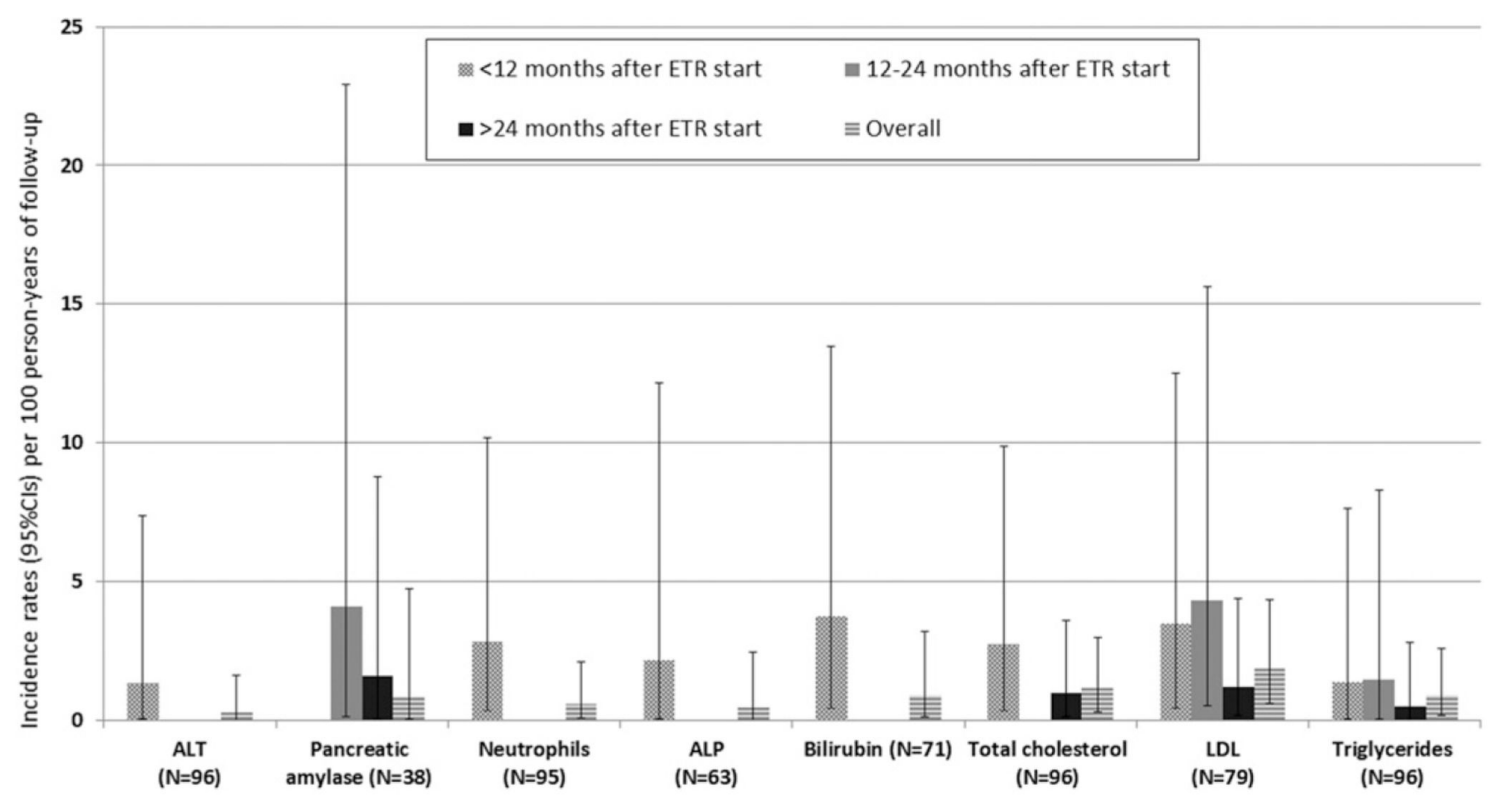
Incidence rates (95%CIs) per 100 person-years of follow-up for DAIDS grade ≥3 laboratory adverse events after ETR start, both overall and by time-period in children 6-<18 years taking a licensed dose (*N*=96 with lab data)

DAIDS grade ≥3 rates of laboratory AEs for the licenced dose group are shown in Figure 1. Rates were highest in the first 12 months on ETR (range, 0.5–4.2 per 100 person-years) and declined thereafter for raised ALT, ALP, bilirubin, total cholesterol, LDL and reduced absolute neutrophil count. For raised pancreatic amylase, rates were highest at 12–24 months and declined thereafter. For raised triglycerides, rates remained relatively constant. No grade ≥3 AEs were reported for raised AST, fasting and non-fasting plasma glucose or pancreatic lipase across all groups (data not shown).

Amongst 138 (78%) patients with clinical data, eight clinical AEs (in eight patients), were reported as possibly, probably or definitely causally related to ETR. All were cutaneous/hypersensitivity reactions occurring within 1 month of ETR start in the licenced group. Two were serious, both Stevens–Johnson Syndrome (SJS), of which one was considered life-threatening. Both patients were on regimens containing ETR and darunavir and were reported as causally related to either drugs. Both events resolved after temporary discontinuation of all ART. The remaining AEs were five rash/erythema and one generalised hypersensitivity/urticaria reaction. Of these, five events resolved (two patients stopped ETR due to the event) and one had unknown resolution status. Amongst patients with clinical data, 5.8% [95%CI 2.7, 10.7] reported skin/hypersensitivity reactions whilst on ETR, of which two (1.4% [95%CI 0.2, 4.7]) were SJS, occurring at a rate of 0.5/100 person-years [95% CI 0.1, 2.1].

Three patients died, two whilst on ETR and one within 3 weeks of stopping. Two were AIDS-associated events and one was an HIV-related metastatic adenocarcinoma. None were considered ETR-related.

By last follow-up 81 patients (46%) discontinued ETR. Median [IQR] time to discontinuation was 23 [8, 47] months, the majority (69%) discontinuing at >12 months. Common reasons for discontinuation were: treatment simplification (19%), treatment failure (16%), toxicity (12%), physician’s decision (10%) and unknown reason (21%) (Table 2). Of those stopping for toxicity, six (50%) were due to hypersensitivities, three (25%) gastrointestinal toxicities and one (1%) lipodystrophy. The proportions, reasons and timing of discontinuation were similar across licencing groups.

## Discussion

The World Health Organization has highlighted the need for more data on outcomes on paediatric second and third-line ART [16]. This cohort study is one of the largest paediatric studies of ETR in a ‘real world setting’ to date and pre-dominately includes highly treatment-experienced adolescents. The majority received ETR combined with PI-based optimised background regimens. Amongst those on ETR at 12 months, nearly 70% were virally suppressed<50 copies/ mL and there was a substantial increase in the median CD4 cell count. These findings are comparable to the PIANO trial, an open-label single-arm ETR trial with optimised background regimen in treatment-experienced children and adolescents aged 6–<18 years (*n*=101) [9]. At 48 weeks, 56% of children and 68% of adolescents had VL<50 copies/ mL and median change in CD4 cell count from baseline was 156 [141, 178] cells/mm^3^. A Spanish observational cohort (*N*=23) of treatment-experienced children/adolescents reported similar findings; 78% had VL<50 copies/mL at a median of 48 weeks [10]. The recent P1090 trial on ETR in treatment-experienced children aged 1–6 years reported that among children on the final dose, 75% of those aged 2–6 years and 33.3% of those aged 1–2 years had VL<400 copies or ≥2 log reduction from baseline at week 48 [14].

Approximately half of the participants in our study discontinued ETR by end of follow-up. The most common reason was treatment simplification reflecting availability of new drugs, including integrase inhibitors [17].

DAIDS grade ≥3 laboratory events occurred infrequently, and clinical AEs were uncommon. Two (1.4%) patients experienced Stevens–Johnson Syndrome, a severe and potentially life-threating hypersensitive reaction. Both were taking darunavir concomitantly and the events were reported as causally related to either/both drugs. Both events resolved after temporary discontinuation of ART. Previous studies have reported associations between both ETR and darunavir and SJS/other cutaneous hypersensitivity reactions [18,19]. The prevalence of SJS in our cohort was higher than that previously reported in adults on ETR (<0.1%) [4].

This study has some limitations. The median duration of follow-up on ETR was relatively short at ∼2 years and we did not have data on HIV drug resistance to assess how this may affect treatment response. However, our study includes data from cohorts across Europe and Thailand and contributes important information on outcomes in children/adolescents on advanced therapy. ETR is now approved for children aged ≥2 years, although there remains scarce data in this younger age group and further monitoring is needed. Paediatric ETR use has some disadvantages including twice-daily dosing, lack of a fixed-dose combination formulation and multiple interactions with other anti-retrovirals [4,8]. Nonetheless, ETR remains an option to combine with optimised background regimens for treatment-experienced children and adolescents.

## Figures and Tables

**Table 1:** Characteristics at etravirine (ETR) start and at 12 months

	*n* (%) or median [IQR]	
*Characteristics at ETR start (n=177)*		
Country		
Spain	71	(40%)
Italy	35	(20%)
Thailand	24	(14%)
UK/Ireland	23	(13%)
Other^a^	24	(14%)
Sex, male	96	(54%)
Ethnic group		
White	63	(36%)
Black African	22	(12%)
Other/unknown	92	(52%)
Mode of HIV acquisition		
Perinatal	173	(98%)
Other/unknown	3	(2%)
Age at ART initiation (years)	2	[0, 5]
Age at ETR start (years)	15	[12, 16]
Treatment status		
Naïve	1	(1%)
Treatment experienced	175	(99%)
Median duration since ART initiation (years)	11	[8, 13]
Triple class exposure (NRTI, NNRTI and PI)	123	(70%)
Prior CDC C diagnosis (*n*=172)	78	(45%)
Median CD4 cells/mm^3^ (*n*=140)	480	[287, 713]
Viral load <50 copies/mL (*n*=143)	28	(20%)
Viral load <400 copies/mL (*n*=143)	73	(52%)
***Dosing/treatment group (n=177)***		
(1) Licenced dose	108	(61%)
(2) Unlicensed dose	10	(6%)
(3) Off-label use, ART inexperienced^b^	7	(4%)
(4) Off-label use, age <6 years	6	(3%)
(5) Missing weight/dose	46	(26%)
***Treatment outcomes at 12 months (n=141)***		
Viral load <50 copies/mL (*n*=124)	85	(69%)
Viral load <400 copies/mL (*n*=124)	99	(80%)
Median CD4 count, cells/mm^3^ (*n*=103)	658	[454, 853]
Median change in CD4 since ETR start (*n*=83)	147	[16, 267]

Note: NRTI; nuclueoside reverse transcriptase inhibitor; NNRTI; non-NRTI; PI: protease inhibitor.

(a) Other countries (number of patients): Belgium (3), Germany (1), Poland (1), Portugal (4), Romania (2), Russia (5), Sweden (1), and Switzerland (7).

(b) Includes seven patients who were categorised as ‘off-label use treatment inexperienced’ as no documented prior exposure to PI or NNRTI drug classes.

**Table 2:** Timing of and reasons for ETR discontinuation.

	*n* (%) or median [IQR]		
Discontinued ETR at last follow-up	81/177	(46)
Time to discontinuation (months)
Median	23	[8, 47]
<1 month	10	(12)
1-5 months	7	(9)
6-11 months	8	(10)
≥12 months	56	(69)
Reasons for stopping ETR		
Treatment simplification	15	(19)
Treatment failure	13	(16)
Toxicity^a^	10	(12)
Physician’s decision	8	(10)
Non-compliance	6	(7)
At patient’s request	6	(7)
Deaths (unrelated to ETR)	2	(3)
Other^b^	4	(5)
Unknown	17	(21)

a Of those stopping for toxicity, six were due to hypersensitivity reactions, three due to gastrointestinal toxicity and one due to lipodystrophy.

b Of those stopping in the ‘other’ category, reasons comprised: one due to availability of a more effective treatment, one due to a change in treatment not due to side effects/poor adherence/contra-indication, one due to ETR becoming unavailable and one as study treatment completed.
